# Parental communication on sexual and reproductive health issues to their adolescents and affecting factors at Asella town, Ethiopia: a community-based, cross-sectional study

**DOI:** 10.1186/s12978-022-01408-8

**Published:** 2022-05-08

**Authors:** Daniel Bekele, Abdi Deksisa, Wondu Abera, Getu Megersa

**Affiliations:** Department of Midwifery, Health Sciences College, Arsi University, Asella Town, Ethiopia

**Keywords:** Adolescent, Communications, Parent, Reproductive health

## Abstract

**Background:**

Parents’ communication on sexual and reproductive health issues with their adolescent plays a great role in preventing morbidity and mortality associated with sexual behavior. However lack of parent to adolescent communication was a serious problem in Ethiopia resulted in teenage pregnancy, unsafe abortions, sexually transmitted infections, school problems, and other sexual risk behaviors. Parents have high responsibility on cultivating their son and daughter regarding to sexual and reproductive health issues. Therefore, the objective of this study was to determine the magnitude of parent’s communication with their adolescents and affecting factors in Ethiopia.

**Methods:**

A community-based, cross-sectional study was conducted on 347 respondents. A systematic sampling method was used to select the study participants. Data were collected by trained interviewers using a structured questionnaire, entered into Epi-Info version 7.1.2 and exported to SPSS version 23 for analysis. Descriptive statistics, bivariate and multivariate logistic regression analyses were used. Variables at *P-value* < 0.05 were considered as significant associations.

**Results:**

Slightly more than one-fifth of the parents (21.3%) had communicated with their adolescents on sexual and reproductive health issues. Associated factors like: being knowledgeable [AOR = 3.08, 95% CI: 1.89–5.39] and being having positive attitudes [AOR 3.03, 95% CI: 1.37–6.70] towards sexual reproductive health issues were significantly associated with communication.

**Conclusion:**

Overall a low proportion of parental communication with their children was identified on sexual and reproductive health issues. This was affected by multidimensional factors to determine their discussion. Thus, promotion of parent to adolescent communication, parents training and addressing the importance of parent to young people communication along with health care providers was important.

## Plain language summary

Parent communication on sexual and reproductive health (SRH) with their adolescent is one of the potential sources of information for adolescents on the issues. Agreed that adolescents in Ethiopia are faced with increasing SRH-related risks, it is important to understand how parents communicate about SRH to their adolescents from the parents’ perspectives. A community based cross-sectional study targeting parents was conducted at eight (8) kebeles of Asella town, Arsi zone, Ethiopia based on an interview survey. Three hundred forty seven (347) parents having children of 10–19 years in the selected household were included. The majority of the parents, 82.4%, agreed on the need for discussions with their adolescents and 72% had a good knowledge of SRH issues. However in this study, the level of parent-adolescent discussion on SRH issues was very low, 23.1%. The find showed that the main reason for not talking with their adolescents; perceived it may initiate adolescent for sexual practice, culturally unacceptable, shame/taboo, lack of awareness and being too busy. Also, the probability of discussion was found to be significantly higher among parents who had completed some form of education, parents who had good knowledge and positive attitude towards SRH issues as compared with those who didn’t have good knowledge and positive attitude on the topic. This study suggests conducting qualitative research investigating the socio-cultural context within which the SRH communications happen.

## Introduction

The World Health Organization (WHO) defines an adolescent as an individual in the age group 10–19 years old [1]. Adolescence is the stage of transition from childhood to adulthood, which is characterized by physiological, psychological and social changes. In addition, adolescence is an occasion to consider health promotion efforts on reducing the risk of negative sexual and reproductive health (SRH) outcomes, such as teen births and sexually transmitted infections (STIs) [1, 2]. Evidences have shown that when adolescents mostly girls communicate to their parents about sexual behaviors, pregnancy prevention (contraception) and STIs they are more likely to engage in safe sexual behaviors, including abstinence and protective behaviors that prevent pregnancy and STIs [2, 3]. In Ethiopia, more than 35% of the total population comprises adolescents. This big number is still suffer from life threatening of sexual and reproductive health (SRH) risks related to early marriage, unwanted pregnancies, unsafe abortions, STIs including HIV/AIDS [4]. Parent-teenager communication may be particularly important, especially when it comes to reducing engagement in sexual risk behaviors [2, 4]. It is believed that, family has the power to guide and encourage teenagers to practice reasonable sexual behavior for good personal decision-making skills [4].

An adolescent who has discussed about their sexual and reproductive health issues were more likely postpone early sexual intercourse and use contraception [5]. Even though parents are the main sources of information for SRH issues, In Ethiopia parental discussion with their adolescents is low and many teenagers do not have access to reliable information on their SRH needs [6]. Furthermore, studies have shown that only 27%, 19%, and 35.0% of parents in Tanzania, Rwanda and Ethiopia, respectively, had communicated about SRH with their adolescents [7–9].

Regarding SRH many adolescents discuss with their peers who may not have a proper knowledge on these matters and as a result gain defective knowledge. This misinformation can make adolescents vulnerable to unprotected sex, unwanted pregnancy, sexually transmitted diseases, and unsafe abortions [10]. Study showed that most of people become sexually active during adolescence [11]. However, the use of contraceptives and condoms among these people are low and unprotected sex is the second largest contributor to health risk in terms of the burden of disease in young people [11]. Globally, each year there are at least hundred million cases of STIs and more than 2.5 million unsafe abortions were reported among adolescent people [12]. In Ethiopia, 13% adolescents face the challenge of early marriage, among which early childbearing is more common in rural than in urban areas 15% and 5%, respectively because of lack of information which could be improved by communication between parents with their adolescents [10, 13]. Consequently, study revealed that teenage mothers are more likely to experience adverse pregnancy outcomes than adults [14].

Parent-adolescent discussions regarding sexuality or SRH issues are more likely to reduce adolescent risk-taking sexual behaviors when combined with effective parent communication with their adolescent of SRH matters [15]. Even though parent-adolescent sexual communication is a primary means of transmitting sexual values, beliefs, expectations, and knowledge between parents and children, proportion of parents who discussed with their adolescent was low [16]. Instead, study showed that the most important sources of information on SRH for adolescents were none family members like friends [17].

Parents have high responsibility on cultivating their children by communicating on their sexual and reproductive health issues. As far as our knowledge is concerned, this is an untapped research area in Ethiopia. Therefore, this cross-sectional study will have a good contribution to the specific field of reproductive health. Hence, this study was conducted to determine parent’s communication with adolescents on SRH issues and affecting factors. The study was used to provide baseline information for policy makers, program planners and implementers to design appropriate interventions to address the SRH issues of adolescents.

## Methods and materials

### Study setting and period

The study was conducted in Asella town, Arsi zone, Ethiopia from May 1 to 30, 2019. Asella is the capital town of West Arsi Zone located 175 km from Addis Ababa. According to the information obtained from the town statistics office report, the total population of the town was 67,269, of whom 33,826 were men and 33,443 were women and had 19,527 households. For the administrative purpose, the town was divided into 8 administrative units (kebele). In the town there are 1 teaching public hospital with 2 health centers and 1 private hospital with one youth clinic family guidance association of Ethiopia (FGAE).

### Study design and population

A community- based cross-sectional study design was conducted quantitatively. All parents/guardians living in Asella town having children of 10–19 years in selected households (HHs) were study populations whereas parents in selected HHs having at least one adolescent during data collection period was considered as study unit. Parents who were severely ill and unable to hear and speak were excluded from the interview. For parents who have male adolescents the fathers/male guardians were interviewed whereas, for female adolescents mother/female guardians were interviewed.

### Sample size determination and sampling procedure

**Fig. 1:**
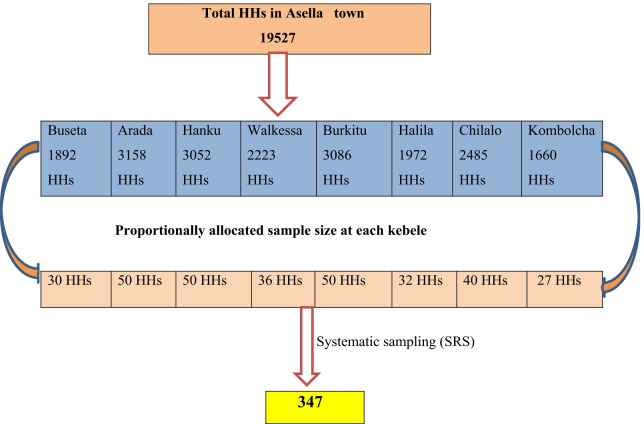
Schematic diagram of sampling technique, 2019

Sample size was calculated using a single population proportion formula with the following assumptions: proportion of parent to adolescent communication 28.76% in previous study in Ethiopia [18], confidence interval 95%, margin of error 5%, and with 10% non-response rate. Accordingly, the total sample size was 347. Asella town has eight (8) Kebeles (smallest administrative unit in Ethiopia). The study was conducted at 8 kebeles with a total of 19,527 households (HHs). Among these eligible 347 HHs were contacted for the study. The number of households to be included in each kebele was determined in proportion to the total number of households for each. Then a systematic random sampling method was used to select the eligible HHs at every Kth interval for each kebeles (Every kth = Total HHs at each kebele /sample size). For those HHs with no eligible candidate (has no adolescents) the interview was conducted in the next HHs where there was an eligible candidate. And also, during data collection the eligible closed households were revisited for interview two times in order to enhance the response rate but for those who failed to get the HHs open (parent not available) the next nearest HHs were interviewed (Fig. 1).

### Data collection procedure and measurement

Trained midwives collected the data by face to face interview using a structured standard questionnaire that was adapted by reviewing relevant literature [18, 21]. To maintain the reliability of the tool the questionnaire was prepared in English and then translated to regional language, Afaan Oromo and back to English by expertise in both languages. The data collectors were trained for 2 days about the objective of the study, the handling of study participants, and other ethical issues.

The questionnaires were pre-tested on 5% of participants out of the study participants 1 week ahead of actual data collection and further refined based on the results. The data was monitored daily during collection. Collected data was checked for completeness and consistency during the interview and at the end of each day. The questionnaire consisted of socio-demographic characteristics, knowledge about SRH, attitude towards SRH, and discussion of SRH related questions. The interview was conducted in a private place and under supervision of the principal investigators.

### Data management and analysis

The data collected through the structured questionnaire was compiled, reviewed, coded and entered into Epi-Info version 7.1.2 and exported to SPSS version 23 for analysis. Data was checked and cleaned for its completeness and errors in coding and entering before analysis. To explain the study population in relation to relevant variables, frequencies tables, graphs and text was used. Dependent variables were computed from responses to SRH communications and were dichotomized as “Yes” (coded as “1”) and “No” (coded as “0”). Then, all variables having *P* value < 0.25 had significant association from binary logistic regression was entered to multiple logistic regression analysis to determine independent associated factor of parent-adolescent communication on SRH issues by controlling the effect of possible confounder, significant statistical association was determined by using AOR at 95% confidence interval(CI) and *P* value < 0.05.

### Operational definition

- *Adolescent* Individual’s teenagers between the age group of 10–19 years old [1].
- *Parents* Biological parents/guardian parent but it does not include elder siblings [11].
- *Communications on SRH issues* Parents/guardian who discussed at least two SRH issues (condom, STIs /HIV/AIDS, sexual intercourse, menstruation, unwanted pregnancy, contraception, physical and psychological changes during puberty) with their adolescents in the last 12 months [11, 18].
- *Knowledgeable on SRH* Parents who scored above summed mean score value of the knowledge questions whereas those who scored below or equal calculated mean value were considered as not knowledgeable[11, 18].
- *Positive attitude* We generated a summated composite score by taking the higher of the communication from the parents where higher indicates greater communication quality and then classified into positive or negative using mean value. Parents who scored above the mean of the attitudinal questions were considered as having a positive attitude while those who scored below or equal the mean value were considered as having a negative attitude [11, 18].

## Results

### Socio-demographic characteristics of respondents

In this study a total of 347 parents were interviewed giving a 100% response rate. The mean age of study participants was 45.9 (± 11 SD) years. As shown in (Table 1) below, among the study participants, 173 (49.9%) were Oromo by ethnic group and 151 (43.5%), of them were orthodox in religion. Majority of the respondents were females 205 (59.1%), regarding marital status majority, 282 (81.3%) was live together and housewives 139 (40.1%), and 128 (36.9%) educated primary school and 162 (46.7%) had (3–5) family size.

**Table 1:** Socio-demographic characteristics of respondents, Asella town, Ethiopia, June, 2019

Variable /categories	Frequency	Percentage
Age		
< 35 years	64	18.4
35–45 years	128	36.9
> 45 years	155	44.7
Sex		
Male	142	40.9
Female	205	59.1
Educational level		
Illiterate	85	24.5
Primary (1–8)	128	36.9
Secondary (9–12)	46	13.3
Diploma and above	88	25.4
Marital status		
Married/ was live together	282	81.3
Divorced	39	11.2
Widowed	26	7.5
Family size		
[1, 2]	122	35.2
[3–5]	162	46.7
> 5	63	18.2
Religion		
Orthodox	151	43.5
Muslim	114	32.9
Protestant	80	23.1
*Others	2	0.6
Ethnicity		
Oromo	173	49.9
Amhara	117	33.7
Gurage	56	16.1
**Others	1	0.3
Occupation		
Housewife	139	40.1
Government employer	73	21
Farmer	60	17.3
Merchant	62	17.9
Daily laborer	13	3.7
Monthly income		
< 500 Ethiopian birr (ETB)	70	20.2
500–1000 ETB	87	25.1
1000 and above ETB	190	54.8

*Others (Wakefata, Adventist and Catholic) **Others could be (Tigire and Wolaita)

### Knowledge on sexual reproductive health issues

**Fig. 2:**
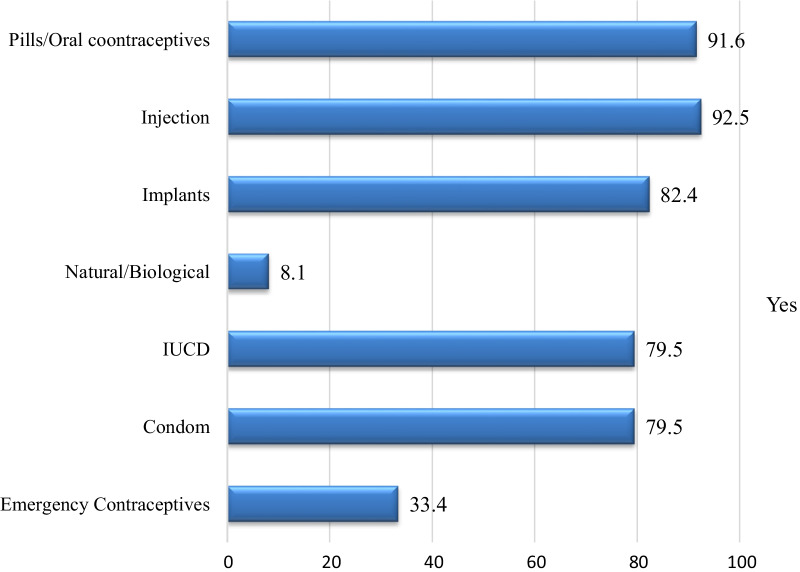
Parents’ knowledge of family planning methods available in Asella town, Arsi zone, Ethiopia, June, 2019

Among the respondents 250(72%) of them were knowledgeable of SRH issues. Specific components of SRH mentioned by the parents were; family planning 93.7%, STDs 73%, and early marriage 52.4%. When asked about the behavioral and physical changes during adolescence, the majority 92.8% breast enlargement and 90.5% beginning of menses on females and change in voice for males 84.7%. Among parents asked about knowledge of contraceptive methods, the majority had awareness of pills 91.6%, injection 92.5%, implant 82.4%, natural /calendar 8.1%, IUCD 79.5%, condom 79.0.5%, and emergency contraceptives 33.4% (Fig. 2). Regarding of the consequences of unprotected sex, the majority reported that leads for STD /HIV 75%, unwanted pregnancy 66%, unsafe abortion 28.2%, and school drop 90.2%.

### Parent’s attitude and suggestions on SRH discussion

**Fig. 3:**
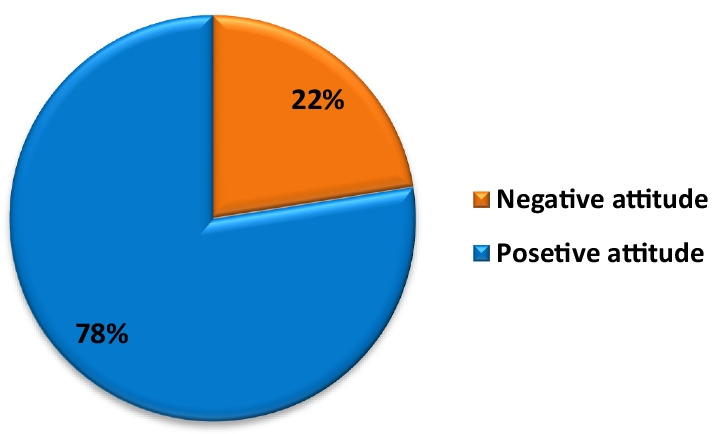
Over all parents attitude of SRH discussion with their adolescents, in Asella town Arsi zone, Ethiopia, June 2019

Attitude of parents towards SRH issues discussion was measured by a set of questions using the likert scale. The majority of the parents 82.4%, agreed on the need of discussions with their adolescents, 89.6% strongly agree to encourage adolescents to ask about SRH information, and 76.4% agreed abstinence of sex rather than other contraceptives. Around 4.3% of parents think that discussion about sexuality will make adolescents promiscuous and 22.8% of parents approve of the use of condoms by their adolescents. In general a combined score for the five questions indicated that 77.5% of parents had a positive attitude towards reproductive health and its discussion (Fig. 3). Among parents asked their suggestions of SRH communication with their adolescents, the majority of parents recommended that adolescents should get adequate information on SRH issues at school 95.4%, through mass media 87%, at home 13.8% and 15% at religious areas. Out of parents asked about their adolescents future sexual behaviors 79.5% were worried about it and 93.4% of them did not accept premarital sex.

### Parent-adolescent communication and hindering reasons on SRH matters

**Fig. 4:**
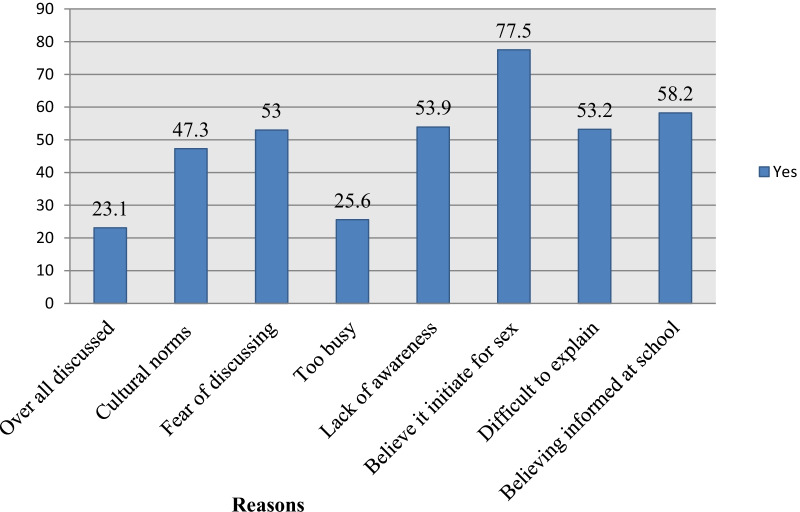
Respondents of overall discussion and reasons for not discussed, in Asella town, Ethiopia June, 2019

In this case participants were asked by a Yes or No question whether they have ever discussed SRH with their adolescents. Even though the majority, 78% of parents, have a positive attitude towards parent-adolescents’ SRH discussion, this study showed that only 23.1% of the respondents had discussed at least two components of SRH issues in the last 12 months. Out of the discussions 60% had been made with their daughters, and 40.3% were done with their son. The finding showed that the majority of female parents prefer to communicate with their daughters 50.7% while male parents have discussed it with sons and daughters, 53.1%. The major topics of the discussions were about STIs/HIV/AIDS 90.2%, abstain 74.2%, early marriage 63.89%, condom 40.02%, and unwanted pregnancy, 40.5%. As seen in (Fig. 4), the most common reason for not talking with their adolescents; majority 77.5%, perceived it may initiate adolescent for sexual practice, culturally unacceptable 47.3%, difficult to explain 58.2%, shame/taboo 53%, lack of awareness 53.9% and lack of time/too busy 25.6%.

### Factors associated with SRH communication

As indicated in Table 2, below. A binary logistic regression analysis showed that parents’ educational status, marital status, family size, attitude and knowledge of parents were significantly associated with parent-adolescent discussion. In multivariate logistic regression all significant variables mentioned above and those with *P-value* less than 0.25 in the crude analysis were again entered into multivariate logistic model to control confounding effect. Hence, the probability of discussion was found to be significantly associated with parents who had completed some form of education: grades 9–12 (AOR = 0.423, 95% CI: 1.062–5.529), diploma and above (AOR = 0.775, 95% CI: 2.034–11.213). Also, parents’ marital status of those who divorced was 69% had lower tendency to discuss SRH issues (AOR = 0.314, 95% CI: 0 0.117–0.842). However parents who were knowledgeable and positive attitude towards SRH issues were almost similarly three times (AOR = 3.086, 95% CI: 1.886–5.395; AOR = 3.034, 95% CI: 1.373–6.704) higher in discussing SRH than their counterparts, respectively.

**Table 2:** Predictors of parental discussions with their adolescents on reproductive health issues, Asella town, Arsi zone, Ethiopia, June, 2019 (n = 347)

Variables		Discussion on SRH issues		COR (95% CI)		AOR (95% CI)	
		Not	Yes				
Educational status	Illiterate	48	37	1		1	
	Primary (1–8)	100	28	0.36	(0.44, 3.95)	1.22	(0.39, 3.77)
	Secondary (9–12)	40	6	0.19	(1.097, 5.50)	0.42	(1.06, 5.53)
	Diploma or above	79	9	0.14	(3.00, 15.24)	0.77	(2.03,11.2)
Marital status	Widowed	198	34	1		1	
	Single/guardian	35	15	2.49	(1.23, 5.05)	2.41	(1.13,5.15)
	Divorced	23	16	4.05	(1.94, 8.44)	3.21	(1.47,7.01)
	Live together	11	15	7.94	(3.36, 18.74)	3.36	(1.26,8.89)
Family size	1–2	87	35	1		1	
	3–5	135	27	0.49	(0.28,.87)	0.64	(0.34, 1.22)
	> 5	45	18	0.99	(0.50, 1.94)	1.08	(0.50, 2.31)
Knowledge	Not knowledgeable	38	59	1			
	Knowledgeable	42	208	3.19	(1.88, 5.39)	3.001	(1.66,5.44)
Attitude	Negative	72	8	1			1
	Positive	70	19	2.44	(1.46, 6.97)	3.03	(1.37,6.70)

Note: COR means for bivariate analysis & AOR means for mulitilogistic regression analysis

COR = Crude Odd Ratio, AOR = Adjusted Odd Ratio

## Discussion

In this study, even though the majority of the parents (82.4%) accepted the importance of communication with their adolescents, the study showed that only 23.1% of parents had discussed at least two topics of SRH issues in the last 12 months. This level of communication is also similar in other studies conducted in Ethiopia [18–20] which revealed that the discussion rarely occurs despite accepting its importance). But it is lower than other findings from Southern Ethiopia and abroad USA [10, 21] and higher than the study conducted in Dera Town [5] all these discrepancies may be due to social-economic, cultural difference and difference in accessing of SRH information. The greatest percentage of parent communication was about STIs/HIV/AIDS which accounts for 90.2%, this finding is similar to other studies conducted in Ethiopia [18, 22]. Different authors argued that most parents are focusing on the negative aspects of SRH rather than working on the preventive aspects. The study shows that, parents who attended higher level and secondary level education were more likely to discuss reproductive health issues with their adolescent children when compared to parents who received non formal education AOR = 2.423 [1.063, 5.529] and AOR = 4.775 [1.062, 5.529] respectively. Findings are in concurrence with other studies, mostly in the country in Gojjam and Hawassa has revealed that adolescents whose mother or father was able to read and write were more likely to communicate SRH issues with their parents than those teenagers’ parents unable to read and write [23]. This may be due to adolescents prefer to discuss with their peers/friends rather than their parents because they think that their parents are not knowledgeable about the subject matter or both parent and adolescents may face challenges because of fearing or embarrassing to communicate about their sexuality.

In this study, among parents asked why they do not communicated about reproductive health issues with their children majority 77.5%, of them perceived that discussing about sexual issues might encourage the children to engage in premarital sex. The finding is proportionately higher than the findings of the studies conducted in the selected region of Ethiopia [5, 18]. Additionally (53.9%) of respondents claimed lack of awareness regarding SRH issues as a reason followed by difficulty to initiate discussion due to fear and shyness (53%). Also, 47.3% of parents worried about their culture/cultural taboos, which is lower as compared to studies conducted in other Sub-Saharan regions [24]. However, it is similar to a study conducted in Ethiopia [18]. The reason might be claimed that most parents are focusing on the negative aspects of SRH rather than working on the preventive aspects. The finding shows that parents who had good knowledge of SRH issues were three times more likely to participate in discussion with their adolescents than their counterparts [AOR = 3.008, (1.662, 5.446)]. This is similar to the study conducted a previous that showed the reason for not discussing SRH issues are parents’ lack of knowledge followed by parents’ lack of communication skills [25].

Regarding communication of contraceptive methods in this study the most frequently discussed between parent and adolescent was about abstinence while the least discussed was about the use of condom. This is similar with study conducted in Ethiopia [9, 10] the reason behind might be thinking of that talking about the utilizations of condoms may initiates the adolescent for sexual practices. In this study, most parents have a positive attitude towards the importance of parent-teen discussion on SRH issues. The find showed that those parents who are more educated have more positive attitude towards SRH issues discussion with their adolescents than the counterpart. Additionally, the majority 95.4% and 87% have suggested that adolescents should get adequate information and knowledge regarding their sexuality and reproductive health issues at school and through mass media respectively. The study recommends that adolescent-parent communication on sexual and reproductive health issues and associated factors helps for policy makers, health care providers and any concerned bodies to design appropriate intervention strategies to tackle young generation reproductive health problems. Information obtained here can be used for planning of intervention programs in different parts of the country.

### Strength and limitation of the study

The current study findings have important theoretical and practical implications for identifying the magnitude of parental communication with their adolescents. However, there are some limitations that are worth and taken into account in the interpretation of the findings. Firstly, lack of qualitative data to complement statistics from quantitative data in the form of close-ended questions. Secondly, the absence of adolescents to be interviewed in the study to understand their view. Future researchers should consider interviewing adolescents so that the validity of a parent’s response could be counter checked and verified against the child’s response.

## Conclusion and recommendation

Parents’ communication with their adolescents was very limited and associated with their marital status, attitudes and knowledge of SRH issues. Our findings identify the need to improve SRH communication of parents to their adolescents. Therefore, the policymakers should involve multiple sectors like health, education and youth services to provide many sources of information to change the negative attitudes of parents towards SRH communication with adolescents. Moreover, parents should discuss SRH issues with young children because they will bear the burden of SRH problems. Further studies should be carried out from the adolescents’ perspective to identify factors that affect the discussion of SRH issues between adolescents and parents. Qualitative research should be more in-depth in relation to the SRH topics discussed between parents and adolescents and the barriers to SRH communication between parents and adolescents.

## Data Availability

All of the main data has been included in the results. In case additional materials with details may be obtained from the corresponding author.

## Abbreviations

AOR: Adjusted odds ratio
CI: Confidence interval
COR: Crude odds ratio
EDHS: Ethiopian demographic and health survey
HHs: Households
HIV/AIDS: Human Immune Virus/Acquired immunodeficiency Syndrome
SPSS: Statistical package for social science
SRH: Sexual and reproductive health
STIs: Sexually transmitted infection
USA: United State of America
WHO: World Health Organization
